# Supplementary material for: Sexual and reproductive health rights: A cross-sectional study of knowledge and practice among the married women of reproductive age residing in Besishahar Municipality, Nepal
Source: PLOS Glob Public Health. 2025 Oct 14;5(10):e0004370. doi: 10.1371/journal.pgph.0004370 (PMC12520395; doi:10.1371/journal.pgph.0004370)
Supplement: S1 Text — (DOCX) [file pgph.0004370.s001.docx]

**Sexual and reproductive health rights: knowledge and practice among the married women of reproductive age residing in Besishahar Municipality, Nepal**

**Sampling Procedure**

| Ward Number | Total number of married women of reproductive age group (18-49 years) | Proportionate sample size based on the total population |
| --- | --- | --- |
| 1 | 833 | $\frac{833}{13432} 342=21$ |
| 2 | 1032 | $\frac{1032}{13432} 342=26$ |
| 3 | 1143 | $\frac{1143}{13432} 342=29$ |
| 4 | 642 | $\frac{642}{13432} 342=17$ |
| 5 | 853 | $\frac{853}{13432} 342=22$ |
| 6 | 950 | $\frac{950}{13432} 342=24$ |
| 7 | 2474 | $\frac{2474}{13432} 342=63$ |
| 8 | 2707 | $\frac{2707}{13432} 342=69$ |
| 9 | 725 | $\frac{725}{13432} 342=19$ |
| 10 | 842 | $\frac{842}{13432} 342=21$ |
| 11 | 1231 | $\frac{1231}{13432} 342=31$ |
|  | Total = 13,432 | Total = 342 |
