# Supplementary material for: Sexual and reproductive health rights: A cross-sectional study of knowledge and practice among the married women of reproductive age residing in Besishahar Municipality, Nepal
Source: PLOS Glob Public Health. 2025 Oct 14;5(10):e0004370. doi: 10.1371/journal.pgph.0004370 (PMC12520395; doi:10.1371/journal.pgph.0004370)
Supplement: S2 Text — (DOCX) [file pgph.0004370.s002.docx]

**Sexual and reproductive health rights: knowledge and practice among the married women of reproductive age residing in Besishahar Municipality, Nepal**

Research Tools

**Group ‘A’ Sociodemographic Information**

| S.N. | Questions | Responses | Skip patterns |
| --- | --- | --- | --- |
| 1. | Age |  |  |
| 2. | Ethnicity | 1. Brahmin 2. Chhettri 3. Janajati 4. Dalit 5. Others |  |
| 3. | Religion | 1. Hindu 2. Buddhist 3. Christian 4. Muslim 5. Others |  |
| 4. | Family type | 1. Nuclear 2. Joint 3. Extended |  |
| 5. | Marriage type | 1. Love marriage 2. Arrange marriage 3. Court marriage 4. Child marriage 5. Others (Specify)…. |  |
| 6. | Education | 1. Illiterate 2. Literate 3. Primary 4. Secondary 5. Higher education |  |
| 7. | Father’s education | 1. Illiterate 2. Literate 3. Primary 4. Secondary 5. Higher education |  |
| 8. | Mother’s education | 1. Illiterate 2. Literate 3. Primary 4. Secondary 5. Higher education |  |
| 9. | Educational level of Mother in law | 1. Illiterate 2. Literate 3. Primary 4. Secondary 5. Higher education |  |
| 10. | Educational level of husband | 1. Illiterate 2. Literate 3. Primary 4. Secondary 5. Higher education |  |
| 11. | Occupational status | 1. Unemployed 2. Service 3. Business 4. House makers 5. Farmer 6. Private sector 7. Others (Specify) |  |
| 12. | Husband’s occupational status | 1. Unemployed 2. Service 3. Business 4. Farmer 5. Private sectors 6. Others (Specify) |  |
| 13. | Number of family members |  |  |
| 14. | Average monthly family income | 1. Below 30 thousand 2. 30 thousand- 50 thousand 3. 50 thousand-1 lakh 4. Above 1 lakh |  |
| 15. | Age at marriage | …………..………..years |  |
| 16. | Duration of marital status | …………..………..years |  |

**Group ‘B’ (Knowledge Related Questionnaire)**

| S.N. | Questions | Response | Skip Patterns |
| --- | --- | --- | --- |
| 17. | What do you mean by sexual and reproductive health?(MR) | 1. To produce baby 2. To maintain healthy life 3. To maintain Physical, Mental, Social and reproductive health 4. To have safe sex life 5. Capacity to reproduce 6. To be free from sexually transmitted infections 7. Others (Specify)……….. |  |
| 18. | What do you mean by sexual and reproductive health rights? (MR) | 1. The right to survival and life. 2. The right to liberty and security of the person. 3. The right to equality and to be free from all form of discrimination 4. The right to freedom of thoughts. 5. The right to privacy and confidentiality 6. The right to information and education 7. Right to have safe sex 8. The right to choose whether or not to marry and build and plan a family. 9. The right to decide whether or when to have children. 10. The right to decide whom to marry. 11. The right to health care and health protection. 12. The right to take part in assembly and political participation 13. The right to be free from torture and ill treatment. 14. Others (Specify)………… 15. Don’t know |  |
| 19. | Do you know about right to obtain education, information, counseling and service relating to sexual and reproductive health? | 1. Yes 2. No |  |
| 20. | What is the appropriate age of marriage for girls? | 1. Below 20 years 2. Above 20 years 3. Others (Specify)…. 4. Don’t know |  |
| 21. | What are the consequences of unsafe sex? MR | 1. Unwanted pregnancy 2. Sexually Transmitted Infections 3. Abortion 4. Others (Specify)…… 5. Don’t know |  |
| 22. | What is the right age for getting pregnant? | 1. Early 20s 2. Late 20s and early 30s 3. Late 30s 4. Others (specify)…. 5. Don’t know |  |
| 23. | What are the right to safe motherhood? | 1. Right to have ANC services 2. Right to choose delivery places 3. Right to have PNC services 4. Health checkup of mother and baby 5. Others (Specify)… 6. Don’t know |  |
| 24. | Do you have idea that having nutritious diet during pregnancy and childbirth is the right of Nepalese women? | 1. Yes 2. No |  |
| 25. | Do you have idea that women should have physical rest during condition of pregnancy and childbirth is the right of Nepalese women? | 1. Yes 2. No |  |
| 26. | What are the sexual and reproductive health morbidity? | 1. Uterine prolapse 2. Infertility 3. Obstetric fistula 4. HIV/AIDs 5. Cervical cancer 6. Breast cancer 7. Gestational diabetes mellitus and hypertension 8. Others (Specify)… 9. Don’t know |  |
| 27. | Do you have idea that women have right to be prevented and treated the reproductive health morbidity? | 1. Yes 2. No |  |
| 28. | Do you know about right to information and services of family planning? | 1. Yes 2. No |  |
| 29. | What is the appropriate years for birth spacing? | 1. Less than 3 years 2. At least 3 years 3. Others (Specify)…. 4. Don’t know |  |
| 30. | Do you know right to have safe abortion and post abortion care at Health Facility? | 1. Yes 2. No | If No go to question no. 32 |
| 31. | What are the conditions for abortion in Nepal? MR | 1. Within 12 weeks of pregnancy at any condition 2. Up to 28 weeks of pregnancy in case of rape or incest 3. At any condition if mother’s health is at risk 4. In case of congenital anomaly at any time 5. Others (specify)……. 6. Don’t know |  |
| 32. | Do you know about the right of protection from sexual abuse, reproductive harms and sexual discrimination? | 1. Yes 2. No |  |
| 33. | Do you know that violation of sexual and reproductive health rights is punishable by law? | 1. Yes 2. No |  |
| 34. | What type of punishment are implemented for violation of sexual and reproductive health rights? | 1. Explaining and asking for an apology 2. Imprisonment 3. Fine 4. Both imprisonment and fine 5. Don’t know |  |

**Group ‘C’ (Practice Related Questionnaire)**

| S.N. | Questions | Responses | Skip pattern |
| --- | --- | --- | --- |
| 35. | Do you receive information, education and counselling about sexual and reproductive health rights? | 1. Yes 2. No |  |
| 36. | From where do you receive these information and education? (MR) | 1. Academic course 2. Health worker 3. Family members 4. Friends 5. FCHVs 6. Mass media (Radio, TV, Facebook) 7. Awareness programs/seminars/trainings 8. Others (Specify)…… |  |
| 37. | Who decided your marriage? | 1. Self 2. Parents 3. Grandparents 4. Family members 5. Others (Specify)…… |  |
| 38. | How many children are there in your family | 1. 0 (No children) 2. 1 3. 2 4. More than 3 | In case of no children go to Q.N.46 (If number of children is 1 then go to Q.N.40) |
| 39. | How much was the birth spacing in between two children of your family? | 1. Less than 3 years 2. 3 years 3. More than 3 years |  |
| 40. | Who decides for the birth of the children in your family? (MR) | 1. Self 2. Husband 3. Both husband and wife 4. Father in law and mother in law 5. Others (specify)….. |  |
| 41. | Did you go for ANC visit?(if there is U5 years children in family) | 1. Yes 2. No |  |
| 42. | Where did you go for delivery (if there is U5 years children in family) | 1. Health institution 2. At home 3. Others (Specify)….. |  |
| 43. | Have you ever utilize PNC services provided by the health workers?(if there is U5 years children in family) | 1. Yes 2. No |  |
| 44. | Did you had enough nutritious diet during your pregnancy and childbirth? | 1. Yes 2. No |  |
| 45. | Did you had enough physical rest during condition of pregnancy and childbirth? | 1. Yes 2. No |  |
| 46. | Do you use any family planning devices? | 1. Yes 2. No | (If “No” go to Q. 50) |
| 47. | Did you get counselling while choosing family planning devices? | 1. Yes 2. No |  |
| 48. | What type of family planning method do you use? | 1. Condom 2. Pills 3. Depo-Provera 4. Implants 5. IUDs 6. Vasectomy 7. Minilap 8. Others (Specify)…. |  |
| 49. | Who decides birth spacing in your family? (MR) | 1. Self 2. Husband 3. Both husband and wife 4. Father in law and mother in law 5. Family members 6. Health workers 7. Others (specify)…. |  |
| 50. | Do you have history of abortion? | 1. Yes 2. No | (If No go to Q.N.52) |
| 51. | What was the reason for your abortion ?MR | 1. Female children 2. No desire for children 3. Health problems of mother/child 4. Others (Specify)…… |  |
| 52. | Do you have any reproductive health morbidity? | 1. Yes 2. No | (If no go to Q.N.56) |
| 53. | Have you get treatment of such morbidity? | 1. Yes 2. No |  |
| 54. | If no, what are the reasons? | 1. Long distance of health institutions from house 2. Hesitancy 3. Refusal from husband for treatment 4. Refusal from family for treatment 5. Having no knowledge of the need for treatment 6. Thinking it is not needed 7. Fear of discrimination in society 8. Behavior of health provider 9. Low quality of services 10. Others (Specify)….. |  |
| 55. | Do you face any discrimination in family, societies and health facilities due to those morbidities? | 1. Yes 2. No |  |
| 56. | With whom do you discuss your reproductive health issues? | 1. No one 2. Husband 3. Family members 4. Friends 5. Health workers 6. Others (Specify).. |  |
| 57. | With whom do you have to take permission to go for your health checkup??(MR) | 1. No one 2. Husband 3. Family member 4. Father in law and mother in law 5. Others (specify)…… |  |
| 58. | Have you receive information about the prevention of sexual and reproductive health morbidities? | 1. Yes 2. No |  |
| 59. | Have you ever participated in mother’s group/ health clubs? If yes, how frequently? | 1. No 2. Every year 3. Every 6 month 4. Every month 5. Others (specify)… |  |
| 60. | In your home who used to manage the economic activities? (MR) | 1. Self 2. Husband 3. Both husband and wife 4. Children 5. Father in law and mother in law 6. Others (Specify).. |  |

MR*Multiple Response
