# Supplementary material for: Sexual and reproductive health rights: A cross-sectional study of knowledge and practice among the married women of reproductive age residing in Besishahar Municipality, Nepal
Source: PLOS Glob Public Health. 2025 Oct 14;5(10):e0004370. doi: 10.1371/journal.pgph.0004370 (PMC12520395; doi:10.1371/journal.pgph.0004370)
Supplement: Attachment — Submitted filename: Response to Reviewers1.docx [file pgph.0004370.s006.docx]

**Author Response to Editor/Reviewer’s Comments**

"PGPH-D-25-00425- Sexual and reproductive health rights: knowledge and practice among the married women of reproductive age residing in Besishahar Municipality, Nepal"

Dear Editor and Reviewers,

PLOS Global Public Health,

Greetings from Nepal,

I would like to extend my sincere gratitude to the editorial and review team for the constructive suggestions and comments in our manuscript. This has undoubtedly helped us shape our manuscript to meet the standards of the global scientific arena. Your valuable suggestions and comments were very meaningful to us. We have gone through all of the comments and have tried our best to rectify them. The point-by-point responses are as follows:

| **Editor's Comments** | **Author's Response** |
| --- | --- |
| Please provide additional details regarding participant consent. In the ethics statement in the Methods and online submission information, please ensure that you have specified (1) whether consent was informed and (2) what type you obtained (for instance, written or verbal, and if verbal, how it was documented and witnessed) | Thank you for your suggestion. The written informed consent was taken from the participants, which is clearly mentioned in the methods section under the subheading ethical statement.  Line: 241-243 |
| In the online submission form, you indicated that "Data will be provided by the corresponding author in a reasonable request".  All PLOS journals now require all data underlying the findings described in their manuscript to be freely available to other researchers, either a. In a public repository, b. Within the manuscript itself, or c. Uploaded as supplementary information. | Thank you for the reminder. We will upload the data file as supplementary information along with the revised manuscript. Supplementary file 6. |

**Reviewer 1**

| **Reviewer's Comments** | **Author's Response** |
| --- | --- |
| **Title** | |
| Consider revising the title to explicitly mention the study design, e.g. “A Cross-Sectional Study of…” | Thank you for your feedback. We have revised the title as: Sexual and reproductive health rights: A cross-sectional study of knowledge and practice among the married women of reproductive age residing in Besishahar Municipality, Nepal. |
| Abstract | |
| The statement “women married at an appropriate age were 2.075 times more likely…” should specify what is meant by “appropriate age.” | We appreciate your feedback and revised the sentence as: Women married at age ≥18 years were 2.075 times more likely to have adequate knowledge of SRHR compared to women who had early marriages (<18 years) (aOR=2.075, 95% CI=1.16-3.69).  Line no. 28-29 |
| The second part of the sentence referring to “which was 1.82 (aOR=1.821, 95% CI=1.02–3.24) in the case of good practice” lacks clarity. Consider rephrasing for better readability and separation of findings on knowledge and practice. | Thank you for your feedback. We have revised the sentence.  Line no. 30-31 |
| Introduction | |
| Line 69: Please include a citation to support the 24.6% unmet need for family planning. | Thank you. We have included the citation.  Line no. 71 |
| Line 71: Similarly, provide a citation for the claim regarding unsafe abortions. | Thank you for the feedback. We have included the citation. Line no. 71 and 74 |
| Methods | |
| Line 142: The authors mention that the study was based on STROBE. Since this is a cross-sectional study, it would be useful to elaborate on how the STROBE guidelines were applied or used to structure the study/reporting. | Thank you for your insightful comment. We agree that elaborating on the use of the STROBE (Strengthening the Reporting of Observational Studies in Epidemiology) guidelines adds clarity and transparency to our reporting. As this is a cross-sectional study, we used the STROBE checklist as a framework to ensure comprehensive and standardized reporting of all relevant sections, including the study design, setting, participant selection, variable definitions, data sources, statistical methods, and interpretation of results. We have now added a brief explanation in the Methods section to clarify how the STROBE guidelines guided the structuring and reporting of our manuscript.  Line no. 145-148 |
| The process of scoring for SRHR knowledge and practices is not clearly described. A detailed explanation is needed in the methods section to support the interpretation of Table 2. | Thank you for pointing this out. We agree that a clearer explanation of the scoring process for sexual and reproductive health rights (SRHR) knowledge and practices is necessary to aid in interpreting the findings presented in Table 2. We have now revised the Methods section to include a detailed description of the scoring criteria and classification process used to assess knowledge and practice levels.  Line no. 211-216  Line no. 221-223 |
| Results | |
| The choice of age groupings (18-33) should be justified. Why not use WHO-standard classifications? Moreover, age ranges in the bivariate analysis table differ from those in the sociodemographic table (Table 1). Please ensure consistency or explain the rationale for the variations. | Thank you for your valuable observation regarding the age groupings. We acknowledge that using WHO-standard classifications can enhance comparability across studies. However, in our analysis, we found that the use of multiple standard age categories resulted in sparse data within some groups. Specifically, the reference category had fewer than 10 observations, which violates a key assumption of logistic regression.  To address this issue and ensure the appropriateness of the regression analysis, we categorized age based on the median age (34 years) of the study participants. This resulted in two age groups: 18–33 years and 34–49 years, which provided more balanced cell counts and met the assumptions required for logistic regression.  Additionally, we have now ensured consistency in age groupings across all tables, including the sociodemographic (Table 1) and bivariate analysis tables. |
| Clarify what is meant by “literate only” under the education variable. Does this refer to individuals who can read/write but have no formal education? | Thank you for your suggestion. Yes, literate only refers to individuals who can read/write but have no formal education. We have made necessary corrections in the manuscript as well. Table 1 |
| The categorization of occupation (services, business, private sector) is ambiguous. Consider merging or redefining these categories for clarity. | Thank you for your concern. We have redefined the categories as government job, business and private sector job. We have revised table 1 as well. |
| How was the poverty line determined in this study? Was a standardized tool such as the Poverty Probability Index (PPI) used? Please describe the criteria or methodology used. | Thank you for the feedback. It refers to the income owned by the family members. Average income less than $1.90 per day is defined as below the poverty line, and equal to or more than $1.90 per day is defined as above the poverty line in reference to the World Bank. Ref no. 15  Line no 198-200  Line no. 263 |

**Reviewer 2**

| **Reviewer's Comments** | **Author's Response** |
| --- | --- |
| Please define the poverty line. | Average income less than $1.90 per day is defined as below the poverty line, and equal to or more than $1.90 per day is defined as above the poverty line in reference to the World Bank.  Line no. 198-200, Ref no. 15 |
| Summarize rows 259-271, 274-292 and 313-327. They are repetitions of tables. | Thank you for your observation. Yes, this is the information from the tables, but these are the highlights of the tables. We think it may not be appropriate to remove all these because a short description of the tables, which contains the highlights of the tables are expected in the published articles.  We would like to clarify that the sections mentioned—rows 259–271, 274–292, and 313–327—serve distinct analytical purposes and are not repetitions of tables.  **Rows 259–271** summarize the results of the bivariate analysis examining associations between knowledge and individual independent variables.  **Rows 274–292** present the findings from the multivariable logistic regression analysis assessing factors independently associated with knowledge.  **Rows 313–327** detail the multivariable logistic regression results for practice, examining its association with various predictors.  Each section provides a summary interpretation of different analyses rather than repeating table content. So, we are expecting your understanding in this matter. |
| Poor or average? What is your criteria? | Thank you for your question. We categorized knowledge as **‘adequate’** and **‘inadequate’** based on the **median value**, as the data did not follow a normal distribution (as assessed by the Kolmogorov Smirnov test). Therefore, instead of using terms like "poor" or "average," we applied a median split to create a binary outcome that is statistically appropriate for non-normally distributed data. |
| Percentages do not need to be included in the discussion. | Thank you for the feedback. We have incorporated your suggestion in the revised manuscript.  Line no. 348, 361, 370, 372, 377, 381, 384, 396, 397, 399, 400 |

We attempted to rectify all comments and suggestions that had been raised by the editors and reviewers. We hope that all the concerns have been addressed. We are very grateful for your time and feedback.

Thank you very much.

With Best Regards

Laxmi Gautam
